# Pneumocystis jirovecii Pneumonia in a Patient With Localized Mycosis Fungoides Not Receiving Immunosuppressive Treatment

**DOI:** 10.7759/cureus.51724

**Published:** 2024-01-05

**Authors:** Ryohei Kudoh, Kosaku Komiya, Ryuichiro Takaki, Atsushi Yokoyama, Kazufumi Hiramatsu, Jun-ichi Kadota

**Affiliations:** 1 Respiratory Medicine and Infectious Diseases, Oita University, Oita, JPN; 2 Respiratory Medicine and Infectious Diseases, Faculty of Medicine, Oita University, Oita, JPN

**Keywords:** cytomegalovirus, immunosuppression, cutaneous t-cell lymphoma, mycosis fungoides, pneumocystis jirovecii pneumonia

## Abstract

*Pneumocystis jirovecii *pneumonia (PCP) is the most common opportunistic infection in patients with human immunodeficiency virus (HIV), but it may develop in patients without HIV, whose immune system is suppressed by anticancer or immunosuppressive agents even when indicating normal counts of CD4+ T cells. Mycosis fungoides (MF) is a primary cutaneous T-cell lymphoma, which is believed not to cause immunosuppressive conditions unless it develops leukosis or metastasis or is treated with anticancer drugs or systemic immunosuppressants. Here, we report a case of PCP in a patient with localized MF not receiving immunosuppressive treatment. The patient, a woman in her 70s, presented with persistent dyspnea. High-resolution computed tomography (HRCT) showed diffuse ground-glass opacities in both lungs. Bronchoalveolar lavage fluid was positive for *P. jirovecii*. Moreover, the cytomegalovirus antigenemia test was positive, whereas tests for anti-HIV and antihuman T-cell lymphotropic virus antibodies were negative. The patient was treated with trimethoprim-sulfamethoxazole, prednisolone, and ganciclovir, which gradually improved the symptoms and diminished diffuse ground-glass opacities on HRCT. This case exemplifies a rare presentation of PCP with mild MF that was not treated with chemotherapy or immunosuppressants. The possible mechanisms for the development of PCP are discussed.

## Introduction

*Pneumocystis jirovecii *pneumonia (PCP) is an opportunistic pulmonary infection that affects patients with cell-mediated immunodeficiency, particularly in human immunodeficiency virus (HIV) infection [1]. PCP may also occur in patients with non-HIV immunocompromised status caused by hematologic malignancy, chemotherapy, or immunosuppressants [2]. In recent decades, the prevalence of PCP among non-HIV patients has remarkably increased with more opportunities for receiving chemotherapy or immunosuppressants for postorgan transplantation or autoimmune diseases [3].

Mycosis fungoides (MF) is the most common type of primary cutaneous T-cell lymphoma (CTCL) and is classified as a non-Hodgkin lymphoma [4,5]. MF is clinically characterized by progression from patches to tumors and histologically by an epidermotropic infiltrate of small- to medium-sized CD4+ T cells [6]. Because of primary cutaneous lymphoma, MF is unlikely to suppress the immune system unless it develops leukosis or is treated with anticancer or immunosuppressive agents [7-9]. We encountered a case of PCP that developed in a patient with mild MF without leukosis and prior treatment with anticancer drugs or systemic immunosuppressive agents. To the best of our knowledge, no cases of PCP in a patient with mild MF receiving no systemic immunosuppressive therapy have been reported, whereas atypical pneumonia and opportunistic infections can occur in some immunocompetent individuals. Here, we describe the clinical course and discuss the possible mechanisms of PCP development in our case.

## Case presentation

A woman in her 70s was referred to our hospital because of persistent dyspnea for two days. She had been treated for MF with phototherapy and local radiation to the skin of her left lower leg 13 years ago. Etretinate administration and topical steroidal treatment were continued during the visit. She had never received anticancer or immunosuppressive drugs for MF or other diseases. No other history suspicious of noninfectious lung diseases, including hypersensitive pneumonia or drug-induced lung injury, was observed.

Physical examination revealed 36.9°C body temperature, 85% percutaneous oxygen saturation (SpO2) without supplemental oxygenation, 120/62 mmHg blood pressure, and 62 beats/min heart rate. Laboratory blood tests showed increased levels of C-reactive protein, white blood cell count, and soluble IL-2 receptor and decreased serum albumin levels (Table 1). Cytological analysis of peripheral blood did not reveal any atypical cells.

**Table 1:** Laboratory results.

Variables	Result	Reference interval
White blood cell, counts/μL	8,980	3,000-7,800
Hemoglobin, g/dL	13.7	10.6-14.4
Serum albumin, g/dL	2.71	4.0-5.2
Blood urea nitrogen, mg/dL	15	7-24
Serum creatinine, mg/dL	0.33	<0.7
Lactate dehydrogenase, U/L	260	120-220
C-reactive protein, mg/dL	6.85	<0.3
Soluble IL-2 receptor, U/mL	2,070	157-474
D-dimer, μg/mL	0.47	<1.0

**Figure 1:**
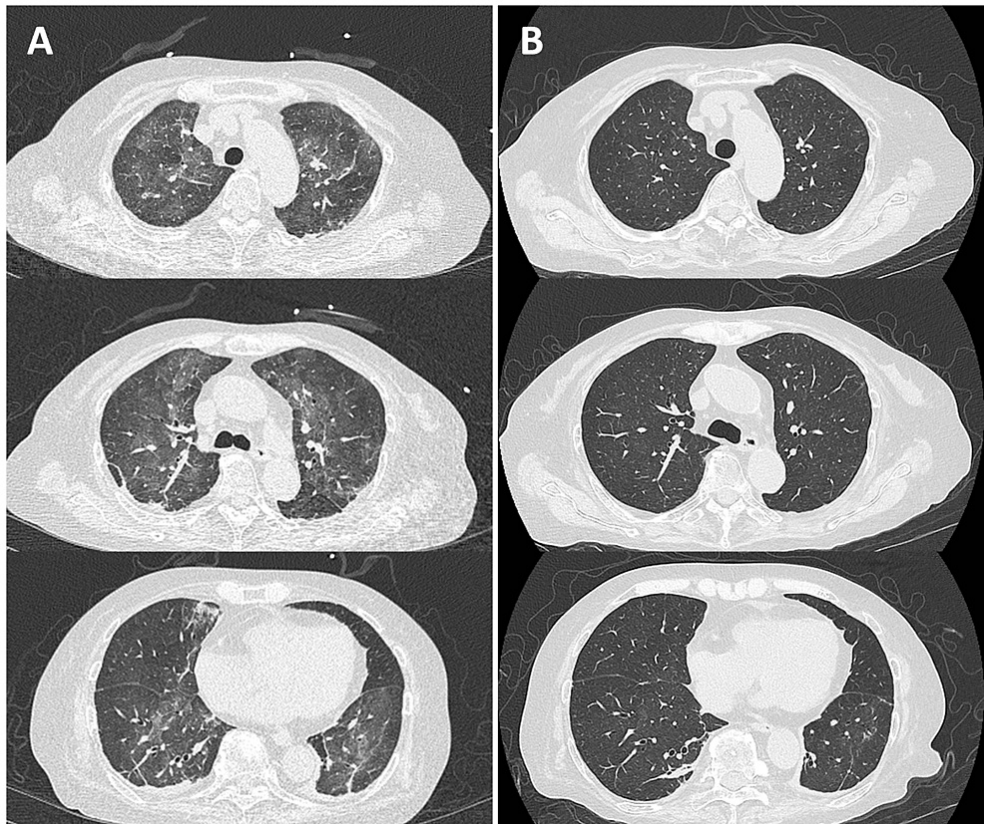
Chest high-resolution computed tomography at the patient’s first visit (A) and 13 days after treatment initiation with trimethoprim–sulfamethoxazole, prednisolone, and ganciclovir (B).

**Figure 2:**
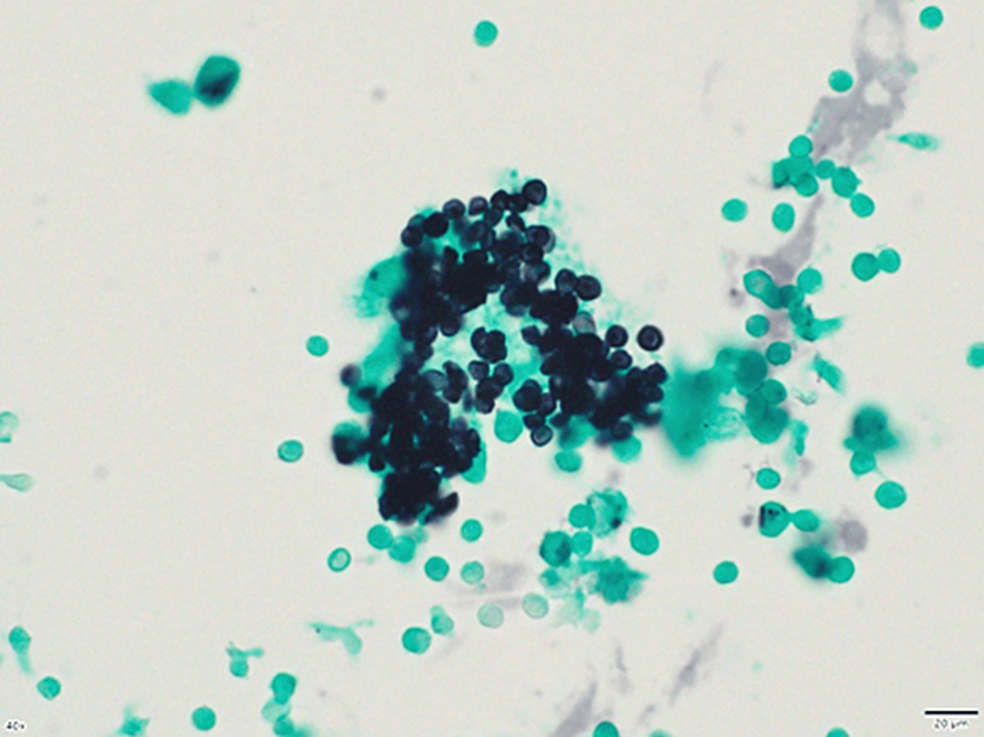
Grocott staining reveals capsular dots in the bronchoalveolar lavage fluid, consistent with Pneumocystis jirovecii infection.

High-resolution computed tomography (HRCT) revealed diffuse ground-glass opacities (GGO) in both lungs (Figure 1A). Polymerase chain reaction (PCR) for severe acute respiratory syndrome coronavirus 2 was negative, but the cytomegalovirus (CMV) antigenemia test (C7-HRP) was positive (46/50000). Beta-D glucan (264 pg/mL) and KL-6 (500.4 U/mL) levels were elevated. Bronchoalveolar lavage fluid (BALF) analysis showed lymphocyte predominance (28.3%), and Grocott’s stain of BALF revealed the presence of *P. jirovecii *(Figure 2), and PCR for *P. jirovecii* using BALF was positive. Although anti-HIV and antihuman T-cell lymphotropic virus 1 (HTLV-1) antibodies were both negative, the CD4+ T-cell count (281/µL) was moderately low.

The patient was ultimately diagnosed with PCP and CMV infection associated with MF. Treatments with trimethoprim-sulfamethoxazole (720 mg/day as trimethoprim) and prednisolone (40 mg/day) for PCP and ganciclovir (500 mg/day) for CMV infection were initiated. Follow-up HRCT 13 days after treatment initiation (day 13) revealed that the GGOs in both lungs diminished (Figure 1B). The patient’s respiratory status improved to not require oxygen supplementation on day 15, and PCP treatment was completed on day 21. Ganciclovir was administered for 22 days until negative confirmation for the C7HRP antigen. The patient was discharged on day 28 with trimethoprim-sulfamethoxazole continued as the prophylactic dose.

## Discussion

The present case was a rare combination of PCP complicated with CMV infection that developed against the background of mild MF with no leukosis or treatment with anticancer or immunosuppressive drugs. Only one case of PCP associated with MF has been reported; however, the patient was treated with systemic glucocorticoids [10]. The possible reasons for the unexpected presentation in the current case need to be discussed.

Several systemic immunodeficiency mechanisms in patients with CTCL are considered. One hypothesis states that antagonization against antitumor immunity induces an immunosuppressive condition. For example, the Jak3/Stat3 pathway is activated in malignant CTCL cells, leading to the production of IL-10 and TGF-β [11-13]. Both cytokines may suppress both cellular and antitumor immunity. IL-10 attacks macrophages, weakens their antigen-presenting ability, and directly suppresses CD4+ T-cell activation [14]. TGF-β inhibits Th1 and Th2 cell differentiation and induces Treg differentiation [15]. The cell surfaces of malignant T cells also express PD-1, PD-L1, and CTLA-4, which act as immunosuppressive proteins [16,17]. PD-L1 binding to PD-1 suppresses T-cell proliferation and production of injurious factors [18]. Furthermore, CTLA-4 binds to CD80 and CD86, competing with CD28 and consequently suppressing T-cell activation [19]. Although clinical evidence of the abovementioned biomarkers is limited, and we did not measure these cytokines in the current case, CTCL may have suppressed the immune system through the mechanisms described, even in mild MF. In fact, the patient’s CD4+ T-cell counts (281/µL) were found moderately low, presumably because of IL-10 production associated with anticancer immunity. The prophylactic administration of trimethoprim-sulfamethoxazole is recommended for patients with HIV having low CD4+ T-cell counts (< 200/µL) or those with PCP history regardless of the CD4+ T-cell counts. While no solid indication for non-HIV patients has been established, our case continued trimethoprim-sulfamethoxazole as the prophylactic dose after the treatment.

In some cases of PCP associated with CTCL other than MF, atypical cells were all found in the peripheral blood, and the condition was diagnosed as leukosis [7,8]. No atypical cells were found in the peripheral blood in the present case; however, the smear test was performed only once. Leukosis complicated with MF is known as Sezary syndrome [4], and repeated blood smear tests or bone marrow biopsy may be required. Similarly, HTLV-1 infection is a known risk for opportunistic infection, but the antibody test was negative in the current case.

## Conclusions

Patients with mild MF who did not receive anticancer or immunosuppressive agents may be susceptible to opportunistic diseases, such as *P. jirovecii *pneumonia or CMV infection. Pneumonia that develops in patients with CTCL needs to be differentiated from those caused by atypical pathogens in addition to noninfectious lung diseases. Measuring cytokines and other markers is advisable to confirm immunosuppression. Furthermore, repeated peripheral blood cytology tests or bone marrow biopsy may be required to monitor leukosis or Sezary syndrome.

## References

[1] Thomas CF Jr, Limper AH (2004). Pneumocystis pneumonia. N Engl J Med.

[2] Salzer HJ, Schäfer G, Hoenigl M (2018). Clinical, diagnostic, and treatment disparities between HIV-Infected and non-HIV-infected immunocompromised patients with pneumocystis jirovecii pneumonia. Respiration.

[3] Maini R, Henderson KL, Sheridan EA, Lamagni T, Nichols G, Delpech V, Phin N (2013). Increasing pneumocystis pneumonia, England, UK, 2000-2010. Emerg Infect Dis.

[4] Larocca C, Kupper T (2019). Mycosis fungoides and Sézary syndrome: an update. Hematol Oncol Clin North Am.

[5] Bradford PT, Devesa SS, Anderson WF, Toro JR (2009). Cutaneous lymphoma incidence patterns in the United States: a population-based study of 3884 cases. Blood.

[6] Stoll JR, Willner J, Oh Y (2021). Primary cutaneous T-cell lymphomas other than mycosis fungoides and Sézary syndrome. Part I: clinical and histologic features and diagnosis. J Am Acad Dermatol.

[7] Kawamoto K, Yamasaki M, Taniwaki M (2021). Smoldering adult T-cell leukemia complicated with pneumocystis pneumonia: a case report. Respir Med Case Rep.

[8] Kunimoto M, Inomata M, Chin H (2023). Adult T-cell leukemia/lymphoma complicated by Pneumocystis pneumonia in a non-endemic area. Respir Med Case Rep.

[9] Kobayashi M, Tsubata Y, Shiratsuki Y, Hotta T, Isobe T (2022). Multiple mass lesions in Pneumocystis pneumonia. Cureus.

[10] Kopterides P, Lignos M, Mavrou I, Armaganidis A (2005). Steroid-induced tumor lysis syndrome in a patient with mycosis fungoides treated for presumed Pneumocystis carinii pneumonia. Am J Hematol.

[11] Kasprzycka M, Zhang Q, Witkiewicz A (2008). Gamma c-signaling cytokines induce a regulatory T cell phenotype in malignant CD4+ T lymphocytes. J Immunol.

[12] Krejsgaard T, Gjerdrum LM, Ralfkiaer E (2008). Malignant Tregs express low molecular splice forms of FOXP3 in Sézary syndrome. Leukemia.

[13] Bagot M, Nikolova M, Schirm-Chabanette F, Wechsler J, Boumsell L, Bensussan A (2001). Crosstalk between tumor T lymphocytes and reactive T lymphocytes in cutaneous T cell lymphomas. Ann N Y Acad Sci.

[14] Brooks DG, Walsh KB, Elsaesser H, Oldstone MB (2010). IL-10 directly suppresses CD4 but not CD8 T cell effector and memory responses following acute viral infection. Proc Natl Acad Sci USA.

[15] Komai T, Okamura T, Yamamoto K, Fujio K (2016). [The effects of TGF-βs on immune responses]. Nihon Rinsho Meneki Gakkai Kaishi.

[16] Kantekure K, Yang Y, Raghunath P (2012). Expression patterns of the immunosuppressive proteins PD-1/CD279 and PD-L1/CD274 at different stages of cutaneous T-cell lymphoma/mycosis fungoides. Am J Dermatopathol.

[17] Wong HK, Wilson AJ, Gibson HM (2006). Increased expression of CTLA-4 in malignant T-cells from patients with mycosis fungoides -- cutaneous T cell lymphoma. J Invest Dermatol.

[18] Okazaki T, Chikuma S, Iwai Y, Fagarasan S, Honjo T (2013). A rheostat for immune responses: the unique properties of PD-1 and their advantages for clinical application. Nat Immunol.

[19] Van Coillie S, Wiernicki B, Xu J (2020). Molecular and cellular functions of CTLA-4. Adv Exp Med Biol.

